# Caregiver’s perceptions of COVID-19 vaccination, and intention to vaccinate their children against the disease: a questionnaire based qualitative study

**DOI:** 10.1097/MS9.0000000000001165

**Published:** 2023-08-09

**Authors:** Farah Yasmin, Kanchan Kumari, Kanza Saleem, Iqra Lareeb, Asim Shaikh, Rija Ashfaq, Bilal Ahmed, Nermeen Bashar, Hala Najeeb, Muhammad Sohaib Asghar

**Affiliations:** a Dow Medical College; b Civil Hospital, Dow University of Health Sciences; c Liaquat National Hospital and Medical College; d Jinnah Postgraduate Medical Centre, Karachi, Pakistan; e Division of Nephrology and Hypertension, Mayo Clinic, Rochester, Minnesota, USA

**Keywords:** adverse event, COVID-19, SARS-CoV-2, side effect, vaccine hesitancy

## Abstract

Coronavirus disease 2019 (COVID-19) vaccine side effects have an important role in the hesitancy of the general population toward vaccine administration. Another reason for vaccine hesitancy might be that healthcare professionals may not address their concerns regarding vaccines appropriately. Regardless, hesitancy in the form of delay, refusal, or acceptance with doubts about its usefulness can limit the downward trajectory of the COVID-19 pandemic. Therefore, the authors conducted a national cross-sectional study (*n*=306) to assess causes and concerns for vaccine hesitancy in caregivers in Pakistan toward getting their children vaccinated. The questions identified caregivers by socioeconomic demographics, perceived COVID-19 pandemic severity, and concerns toward the COVID-19 vaccine. The majority of the participants were 45–59 years of age (42.8%) with a mean age of 36.11 years (SD: 7.81). A total of 80% of these participants were willing to vaccinate their child with any COVID-19 vaccine. Present comorbidities had a frequency of 28.4% (*n*=87/306) and only 26.9% (*n*=66/245) participants were willing to vaccinate their child. Participants with high social standing were 15.4% (*n*=47/306) with the majority of them being willing to vaccinate their children (45/47). Socioeconomic status (OR:2.911 [0.999–8.483]), and the child’s vaccinations being up to date (OR:1.904 [1.078–3.365]) were found to be independent factors for caregivers to be willing to vaccinate their child. Around 62% (*n*=191/306) were not willing to vaccinate due to the concern for side effects, 67.6% (*n*=207/306) were not willing because they did not have ample information available, and 51% (*n*=156/306) were not willing as they were concerned about vaccine effectiveness. Further studies on vaccine safety in the pediatric population are required to improve caregivers’ perceptions.

## Graphical abstract

**Figure:**
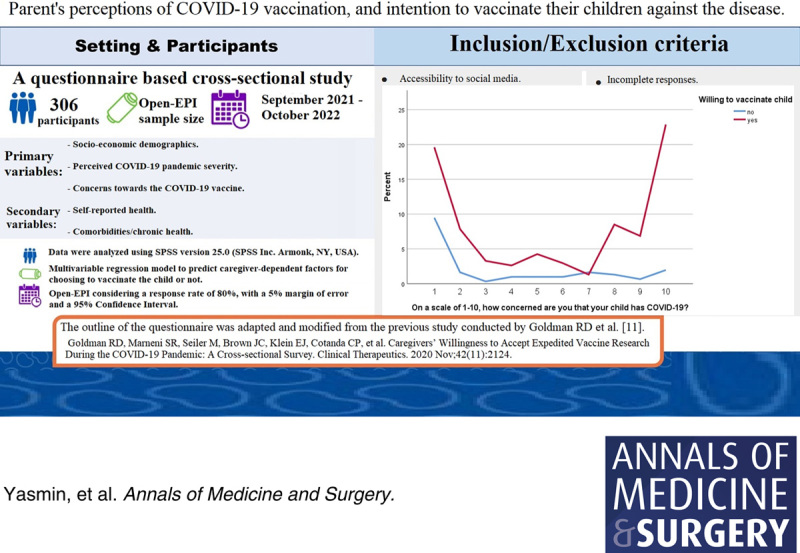

## Introduction

Highlights

- Our study results showed that 80.0% of the studied population were willing to vaccinate their child.
- The top reason to vaccinate is the protection of the child and not to vaccinate is side effects.
- The most significant predictors were high social status and the child’s vaccination being up-to-date.

The coronavirus otherwise known as severe acute respiratory syndrome coronaviruses including SARS-CoV and SARS-CoV-2, first appeared in China^1^. Despite the extensive research performed on SARS, there is limited understanding of the pathophysiological impact of coronavirus disease 2019 (COVID-19). Research shows that SARS‐CoV‐2 causes acute pneumonia with clinical symptoms similar to those reported for SARS‐CoV and MERS‐CoV^2^. In regard to SARS-CoV-2 infection (COVID-19), the most concerning complication to date is acute hypoxemic respiratory failure, which requires patients to be on mechanical ventilation.

As of mid-March 2022, more than 960 000 U.S. residents have died of COVID-19^3^; however, the true number of deaths resulting from COVID-19, both directly and indirectly, is likely to be much higher. According to the recent Center of Disease Prevention and Control (CDC) data, 1078 children and adolescents less than 18 years of age died from COVID-19^4^. As a result, children could benefit both directly and indirectly from the COVID-19 vaccination^5^. Surveys have shown that the rate of hospitalizations and deaths were markedly reduced by 65 and 69% in the U.S., respectively^5^. There is a lack of argument on whether the vaccination rate among children is lower or greater than adults. Despite vaccine introduction, hesitancy in the form of delay, refusal, or acceptance with doubts about the usefulness or its impact against COVID-19 can limit the downward trajectory of the COVID-19 pandemic^6^. Vaccine hesitancy stems from fear and mistrust toward healthcare services and is recognized by the WHO as one of the top 10 most important threats during the pandemic^7^. Even though there is a clear public perception of the high-risk associations regarding the pandemic, many studies show that subgroups around the world are reluctant about vaccination mainly because of its side effects and the conspiracy theories surrounding it. In many countries; however, a considerable fraction of healthcare workers is influenced by vaccine hesitancy due to a lack of information of its safety and long-term efficacy^8^. At the end of the day, most healthcare professionals are not experts on COVID-19 vaccination because of disease novelty that is still under extensive research. They may face a dilemma on how transparent they should be about the risk and benefits of vaccines to individuals without undermining their confidence in an important public health measure.

Another reason for vaccine hesitancy might be that healthcare professionals may not address their concerns regarding vaccines appropriately. This remains the only significant factor despite full access to vaccine services possibly due to fear and mistrust toward healthcare services^9^. COVID-19 vaccine hesitancy perhaps stems from similar reasons. Despite the uncertainties about its risk and benefits, healthcare professionals strongly support COVID-19 vaccination considering its impact in reducing disease mortality and hospitalizations.

Vaccine hesitancy, most importantly, is derived from the exposure to criticism of vaccination, misinformation, and ‘anti-vax’ activists through social networks^10^. False and misleading information about COVID-19, potentially dangerous treatments, and eventual vaccination continue to grow on social media platforms. As the number of people reluctant to get vaccinated is increasing rapidly, with many downright refusing to get vaccinated in the midst of a global pandemic, it is getting increasingly important to understand the causes of vaccine hesitancy to combat it and identify solutions that ameliorate people’s concerns regarding vaccinations in a healthy and cooperative way. Therefore, we conducted a national cross-sectional study to assess causes and concerns for vaccine hesitancy in caregivers in Pakistan toward getting their children vaccinated.

## Material and methods

### Study’s general characteristics

A qualitative study was conducted amongst caregivers with children in Pakistan between September 2021 and October 2021. Three hundred and fifty individuals representing each of the four provinces of Pakistan, Sindh, Punjab, Khyber Pakhtunkhwa, and Baluchistan between the ages of 18 and 69 years provided consent for participation. Research protocol approval was taken from the local registry and carried out according to qualitative study protocols^11^. The sample size was calculated through the WHO sample size calculator by estimating a population proportion with specified absolute precision^12^, using the formula =*Z* ^2^ *P*(1−*P*)/*d* ^2^ where *n*=sample size; *Z*=CI; *P*=anticipated prevalence, and *d*=absolute precision.

An online questionnaire was administered through popular social media platforms such as WhatsApp, Facebook, and via e-mail that was completed by each participant individually. Bloggers and social media influencers were propagating the questionnaire to caregiver’s who consented via online written authorization to access the questionnaire. The consent was provided before responding to the actual questionnaire. Anonymity was part of consenting authorization, and was filled up by the responders. The outline of the questionnaire was adapted and modified from the previous study conducted by Goldman *et al*.^13^ and consisted of several multiple-choice questions as well as questions requiring a yes or no response. The survey tool was translated into Urdu and the questionnaires were provided and subsequently completed between dates. The participants were informed that their data would be anonymous and that it would be kept confidential to minimize response bias. Out of 350 individuals, 306 were selected for final analysis. Results were presented according to guidelines set by strengthening the reporting of cohort, cross-sectional and case–control studies in surgery (STROCSS) group^14^.

### Questionnaire details

The objective of the questionnaire was to investigate predictors associated with Pakistan caregivers’ intent to vaccinate their children against COVID-19. Questions identified caregivers by socioeconomic demographics, perceived COVID-19 pandemic severity, and concerns toward the COVID-19 vaccine. Additionally, questions were included to investigate the age of the caregiver, whether the individual and their child had been vaccinated against influenza and maintained up-to-date records. Participants were also questioned regarding their self-reported health and comorbidities as well as their child’s. Furthermore, it was specifically documented if the child had a chronic illness or if they were using any chronic medications.

### Statistical analysis

Data were analyzed using SPSS version 25.0 (SPSS Inc.). Frequencies were calculated for all categorical responses. A multivariable logistic regression model was used using the odds ratio to predict caregiver-dependent factors for choosing to vaccinate the child or not with dependent variables being the caregiver’s willingness to vaccinate their child. A *P*-value of <0.05 was considered to be statistically significant.

## Results

Out of the total 306 participants, most of the participants were in the age group of 40–59 years (42.8%) and women (79.7%). A total of 80% (*n*=245/306) of these participants were willing to vaccinate their child, and 20% (*n*=61/306) were not. 79.7% (*n*=244/306) participants were female, and out of them 78% (*n*=191/245) were likely to get their child vaccinated, and 86.9% (*n*=53/61) were not. By contrast, 20.3% (*n*=62/306) participants were male with 22% (54/245) of them willing to vaccinate their child as opposed to 13.1% (8/61) that were not.

About 1.3% (*n*=4/306) of the participants had no formal education, 2.6% (*n*=8/306) had primary education, 5.9% (*n*=18/306) had secondary education, 28.8% (*n*=88/306) had higher secondary education, 38.2% (*n*=117/306) of the participants were university graduates, and 23.2% (*n*=71/306) were of postgraduate or higher level. 10.5% (*n*=32/306) participants worked in the public sector, and 9.5% (*n*=29/306) were healthcare workers. 19.3% (*n*=59/306) were private business employees, 25.2% (*n*=77/306) were students, and 35.6% (*n*=109/306) were unemployed. 11% of government workers were willing to vaccinate their child while 5% were against it. 66% (*n*=202/306) of the participants were married with 66.9% (*n*=164/245) of them willing to get their child vaccinated and 29.4% (*n*=90/306) were single, out of which 28.2% (*n*=69/245) were willing to get their child vaccinated. Of the total vaccinated participants, 6.9% (*n*=21/306) earned less than 20 000 PKR monthly, 14.1% (*n*=43/306) earned 20 000–50 000 PKR, 26.8% (*n*=82/306) earned 50 000–100 000 PKR, 25.5% (*n*=78/306) earned 100 000–200 000 PKR, and 26.8% (*n*=82/306) earned more than 200 000 PKR. Participants who made more than 200 000 PKR had a 28.6% willingness to vaccinate their child. 41.2% (*n*=126/306) vaccinated participants were from Sindh, out of which 41.6% (*n*=102/ 245) were willing to get their children vaccinated. Participants from Punjab were 45.8% (*n*=140/306) and 44.9% (*n*=110/245) of them were willing to get their children vaccinated. 11.4% (*n*=35/306) of participants in KPK were vaccinated and 11.8% (*n*=29/245) of them were willing to get their children vaccinated.

Present comorbidities had a frequency of 28.4% (*n*=87/306) and only 26.9% (*n*=66/245) participants were willing to vaccinate their child. 16% (*n*=49/306) of participants had self-reported excellent health, 52% (*n*=159/306) had reported good health, 30.1% (*n*=92/306) reported fair health, and 2% (*n*=6/305) had reported poor health. Participants with high social standing were 15.4% (*n*=47/306), with the majority of them being willing to vaccinate their children (45/47). 64.7% (*n*=198/306) of the children had their vaccinations up to date and 35.3% (*n*=108/306) did not. 16% (*n*=49/306) of them were vaccinated against influenza and 84% (*n*=257/306) were not. The complete breakdown by demographics is shown in Table 1.

**Table 1:** Social and demographic characteristics of the study population

			Willingness to vaccinate their child against COVID-19		
Variables	Characteristics	Frequency (%)	Yes (*n*=245)	No (*n*=61)	Significance
Age	18–29 years	111 (36.3)	84 (34.3)	27 (44.3)	0.231
	30–39 years	50 (16.3)	38 (15.5)	12 (19.7)	
	40–59 years	131 (42.8)	110 (44.9)	21 (34.4)	
	>60 years	14 (4.6)	13 (5.3)	1 (1.6)	
Sex of caregiver	Female	244 (79.7)	191 (78.0)	53 (86.9)	0.121
	Male	62 (20.3)	54 (22.0)	8 (13.1)	
Educational status	No formal education	4 (1.3)	3 (1.2)	1 (1.6)	0.645
	Primary education	8 (2.6)	6 (2.4)	2 (3.3)	
	Secondary education	18 (5.9)	14 (5.7)	4 (6.6)	
	Higher secondary	88 (28.8)	66 (26.9)	22 (36.1)	
	University graduate	117 (38.2)	97 (39.6)	20 (32.8)	
	Postgraduate or higher	71 (23.2)	59 (24.1)	12 (19.7)	
Occupation	Public sector	32 (10.5)	27 (11.0)	5 (8.2)	0.140
	Healthcare workers	29 (9.5)	24 (9.8)	5 (8.2)	
	Private business	59 (19.3)	53 (21.6)	6 (9.8)	
	Students	77 (25.2)	56 (22.9)	21 (34.4)	
	Unemployed	109 (35.6)	85 (34.7)	24 (39.3)	
Marital status	Married	202 (66.0)	164 (66.9)	38 (62.3)	0.643
	Single	90 (29.4)	69 (28.2)	21 (34.4)	
	Widowed/Divorced	14 (4.6)	12 (4.9)	2 (3.3)	
Monthly income	<20 000 PKR	21 (6.9)	15 (6.1)	6 (9.8)	0.428
	20 000–50 000 PKR	43 (14.1)	34 (13.9)	9 (14.8)	
	50 000–100 000 PKR	82 (26.8)	62 (25.3)	20 (32.8)	
	100 000–200 000 PKR	78 (25.5)	64 (26.1)	14 (23.0)	
	>200 000 PKR	82 (26.8)	70 (28.6)	12 (19.7)	
Region	Sindh	126 (41.2)	102 (41.6)	24 (39.3)	0.934
	Punjab	140 (45.8)	110 (44.9)	30 (49.2)	
	KPK	35 (11.4)	29 (11.8)	6 (9.8)	
	Balochistan	5 (1.6)	4 (1.6)	1 (1.6)	
Comorbidities	Present	87 (28.4)	66 (26.9)	21 (34.4)	0.246
	Absent	219 (71.6)	179 (73.1)	40 (65.6)	
Self-reported health	Excellent	49 (16.0)	39 (15.9)	10 (16.4)	0.785
	Good	159 (52.0)	129 (52.7)	30 (49.2)	
	Fair	92 (30.1)	73 (29.8)	19 (31.1)	
	Poor	6 (2.0)	4 (1.6)	2 (3.3)	
Socioeconomic status	High	47 (15.4)	43 (17.6)	4 (6.6)	**0.044**
	Middle	244 (79.7)	192 (78.4)	52 (85.2)	
	Low	15 (4.9)	10 (4.1)	5 (8.2)	
Child’s sex	Female	227 (74.2)	182 (74.3)	45 (73.8)	0.934
	Male	79 (25.8)	63 (25.7)	16 (26.2)	
Child having chronic illness	Yes	22 (7.2)	19 (7.8)	3 (4.9)	0.326
	No	284 (92.8)	226 (92.2)	58 (95.1)	
Child on chronic medications	Yes	16 (5.2)	14 (5.7)	2 (3.3)	0.348
	No	290 (94.8)	231 (94.3)	59 (96.7)	
Relationship with child	Mother	200 (65.4)	158 (64.5)	42 (68.9)	0.310
	Father	55 (18.0)	48 (19.6)	7 (11.5)	
	Guardian	51 (16.7)	39 (15.9)	12 (19.7)	
Child’s vaccination up to date	Yes	198 (64.7)	166 (67.8)	32 (52.5)	**0.025**
	No	108 (35.3)	79 (32.2)	29 (47.5)	
In the last 12 months, was your child vaccinated against influenza?	Yes	49 (16.0)	41 (16.7)	8 (13.1)	0.490
	No	257 (84.0)	204 (83.3)	53 (86.9)	
In the last 12 months, were you vaccinated against influenza?	Yes	43 (14.1)	39 (15.9)	4 (6.6)	0.060
	No	263 (85.9)	206 (84.1)	57 (93.4)	

COVID-19, coronavirus disease 2019; KPK, Khyber Pakhtunkhwa; PKR, Pakistani Rupee.

The willingness to vaccinate children was calculated using the multivariable logistic regression analysis. Two factors were found to be statistically significant in predicting the willingness to vaccinate children in parents/legal guardians, including higher socioeconomic status (OR=2.911, *P*=0.050, 95% CI=0.999–8.483), and the child’s vaccinations being up to date (OR=1.904, *P*=0.027, 95% CI=1.078–3.365). The complete breakdown of the multivariable regression model is shown in Table 2.

**Table 2:** Multivariable regression for predictors of caregiver’s willingness to vaccinate their child

Variables	OR	95% CI	*P*
Caregiver being female sex	0.534	0.239–1.191	0.125
Caregiver’s age between 40 and 59 years	1.684	0.890–3.184	0.109
Caregiver’s education of university graduate	1.617	0.818–3.196	0.167
Caregiver’s occupation as private business	1.765	0.957–5.281	0.062
Caregiver’s marital status as being single	0.761	0.417–1.391	0.375
Caregiver’s household income >200 000 PKR	1.882	0.851–4.159	0.118
Caregiver belongs to province KPK	1.529	0.503–3.468	0.576
Caregiver having no comorbidities	1.424	0.782–2.591	0.247
Caregiver’s self-reported health being poor	0.465	0.081–2.659	0.389
Caregiver’s high socioeconomic status	2.911	0.999–8.483	**0.050**
Child’s sex being female	1.027	0.542–1.945	0.934
Child having no chronic illness	0.615	0.176–2.150	0.447
Child not no chronic medication	0.559	0.124–2.529	0.450
Caregiver being child’s father	1.823	0.769–4.320	0.173
Child’s vaccination being up-to-date	1.904	1.078–3.365	**0.027**
Child took influenza vaccine in past 12 months	1.331	0.589–3.010	0.491
Caregiver took influenza vaccine in past 12 months	2.698	0.925–7.865	0.069

COVID-19, coronavirus disease 2019; KPK, Khyber Pakhtunkhwa; PKR, Pakistani Rupee.

**Figure 1:**
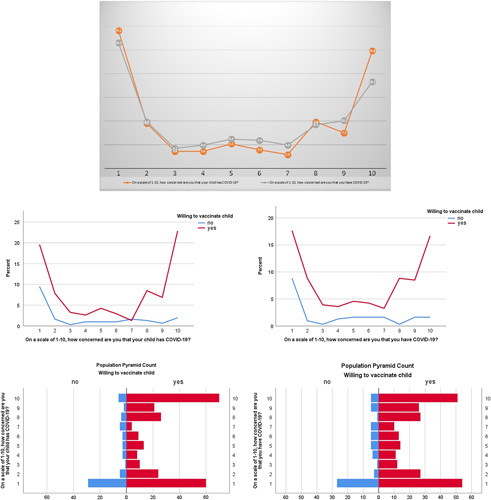
Population pyramid of the distribution scale of 1–10 on willingness to vaccinate children and parental concerns.

The reasons for these inclinations were reported as 97.1% (*n*=297/306) participants were willing to vaccinate their child to protect them, 93.8% (*n*=287/306) were willing to do so their children could return to a normal life and 96.1% (*n*=294/306) were vaccinating their children to protect others. Whereas 62.4% (*n*=191/306) were not willing to vaccinate due to the concern for side effects. 67.6% (*n*=207/306) were not willing because they did not have ample information available. Lastly, 51% (*n*=156/306) were not willing to vaccinate their children as they were concerned about the efficiency and effectiveness of the vaccine, as shown in Table 3. Figure 1 showcased scaled responses of the studied population regarding the vaccine hesitancy.

**Table 3:** Reasons quoted by caregivers for willingness to vaccinate or not to vaccinate their child

Reason to vaccinate	Frequency	Percentage
Protect child	297	97.1
Protect others	294	96.1
Vaccine acceptance	206	67.3
Perceived pandemic severity	255	83.3
Child on high risk for acquiring infection	194	63.4
Accepting but concerned about safety/efficacy	263	85.9
Desire to return to normal life	287	93.8
No comments	95	31.0
Reason not to vaccinate		
** **Novelty	103	33.7
** **Perceived child not at risk for acquiring infection	120	39.2
** **Concerned about side effects/safety	191	62.4
** **May vaccinate if more information available	207	67.6
** **Refusal to vaccination	76	24.8
** **Concerned about efficacy	156	51.0
** **Perceived contraindication	99	32.4
** **No comments	99	32.4

## Discussion

Our primary finding was that higher socioeconomic status predicts a higher willingness to vaccinate children for COVID-19 as well as having an up-to-date vaccination portfolio. This finding is like the results found by multiple other studies^15–17^ where high income and education levels were associated with lower vaccine hesitancy. Medical individuals can better help them understand certain medical procedures and pharmacological interventions such as vaccines as well as help dispel any misinformation or biases that they may hold^18^. However, this finding can be further complicated as recent studies^19,20^ have found that the type of media resource being used to conduct research, especially concerning vaccines can be extremely important in directing decision-making regarding vaccination hesitancy as well as general trust in the national healthcare system. One of the greatest impacts promoting vaccine hesitancy, especially in the West, has been had by social media networks owing to the rampant and quick circulation of articles, curiosity-inducing headlines and sensational journalism. Social media has been implicated as being the biggest propagator of misinformation regarding vaccines and their safety and efficacy. With abysmal rates of fact-checking that occurs on social media, shockingly, the current distrust rates concerning vaccines and medicine in general stand at an extreme high of 48% of individuals not being very likely to accept the COVID-19 vaccines in 2021^21^. A clear but difficult and long-term solution to increasing vaccine acceptance, trust in the healthcare system and improving willingness to readily accept medical interventions seems to be improving the socioeconomic status of the general population; however, that requires a global economic approach.

Our secondary finding was that individuals with children who had up-to-date vaccination profiles were very likely to be accepting of the COVID-19 vaccines. This finding makes complete sense as these children very likely belong to families who are already of a higher socioeconomic status, as mentioned above, or have trust in the healthcare system as well as understand that popular social media claims about medical practices involving vaccinations are not to be trusted and should be thoroughly verified. This finding is in conjunction with the discoveries made by another study where vaccination history was found to be a leading predictor for COVID-19 vaccine acceptance^22^.

We also found that the leading causes for willingness to vaccinate were to protect the child, followed by protecting others. Perceived severity and the noble notion of protecting the wider community have previously proven to be important causes of vaccine acceptance^23,24^. However, age and sex were also found to be important predictive factors by the aforementioned studies. Our study found no statistical significance regarding age and sex as being predictive. An important cultural reason for this is the highly patriarchal nature of society in Pakistan, where fathers and grandfathers make most family decisions, including but not limited to medical decisions; in many cases, one would be correct in assuming that the dominant male figure in the household decides whether the children as well as the women would be receiving medical interventions or not^25,26^ if such is the case, then there would be an absence of differing opinions within families and no sex or age effect would be recognizable within the statistical analyses.

In addition to this, the two most common reasons for not vaccinating were found to be a lack of information and concerns regarding safety and side effects. The lack of information is a relatively simple problem with an easy solution. Physician counseling of patients and individuals is usually the best way to aid in this difficulty. We recommend premade information pamphlets where the most common questions are answered in an easy-to-understand language translated into the multiple languages that people in Pakistan tend to speak. These booklets can then be spread enmasse with the national COVID-19 helpline, which currently exists, and the contact details contained within it. This could perhaps hasten the process of spreading correct and relevant information. However, the former obstacle is the hardest to tackle. Safety is by far the globally leading cause of refusing medical intervention at any and all levels^27^. A patient-centered approach with complete delivery of medical knowledge regarding the intervention, the vaccine in this case, and verification of informed consent, while a consistently implicated, and the most important part of medical ethics, has not yet seemed to solve this problem. Furthermore, with individuals refusing to come to the hospital and not seeking out medical care or the consultation of a medical professional, information and counseling becomes even more difficult to provide. Figuring out the solution to this question seems to be the most baffling yet the most necessary part of the equation during a global pandemic. One thing is for certain; however, that the solution cannot be unilateral. A consistent, tireless, and multivariate approach is needed by healthcare professionals, the elected body of Pakistan and relevant countries, social media companies, and news organizations. Several strategies have been offered consisting of expert fact-checking, verified content tags on social media articles and careful and consistent dissemination of hyperbolic yet misleading content regarding medical literature^28^. This has to be coupled with a publicly funded mass media campaign in developing nations like Pakistan, where access to popular social media platforms like Twitter and Facebook is still limited, and the root cause of hesitancy is actually via popular yet cheap mass messaging platforms like WhatsApp instead.

Our study had some considerable limitations. Firstly, we could not evaluate the proportion of the effect social media platforms had on vaccine hesitancy in individuals. Secondly, we did not evaluate the effect religious and cultural practices had on refusing vaccination, which is extremely important in a country like Pakistan where decision-making is greatly impacted by traditional norms and religious beliefs.

## Conclusion

The current study supports previous medical literature regarding socioeconomic status and vaccination portfolio status being the most important predictive tools when evaluating vaccine hesitancy. We also identified extremely common reasons for both vaccinating and choosing not to do so. Fear of side effects remains a leading cause of refusal to vaccinate, which has to be tackled quickly yet with great care and consideration. A global, combined public and private effort is needed, which cannot be accomplished without some serious oversight and regulations, especially when it comes to dispelling misinformation regarding medical interventions like vaccines.

## Ethical approval

Institutional Review Board – Dow University Hospital (IRB/DUH/2021/789).

## Consent

Content to participate was taken from all of the study participants in a written, informed manner.

## Sources of funding

This work is not supported by any sponsors. No funding is required in this study.

## Author contribution

List of all authors: F.Y., K.K., K.S., I.L., A.S., R.A., B.A., N.B., H.N., M.S.A. Corresponding author: M.S.A. This statement is to certify that all authors have seen and approved the manuscript being submitted, have contributed significantly to the work, attest to the validity and legitimacy of the data and its interpretation, and agree to its submission to the *Annals of Medicine and Surgery*. We attest that the article is the authors’ original work, has not received prior publication and is not under consideration for publication elsewhere. We adhere to the statement of ethical publishing as appears in the journal statement on ethical standards in publishing scientific articles in the IJS publishing group. On behalf of all co-authors, the corresponding author shall bear full responsibility for the submission. Any changes to the list of authors, including changes in order, additions, or removals, will require the submission of a new author agreement form approved and signed by all the original and added submitting authors.

## Conflicts of interest disclosure

The authors declare that they have no conflicts of interest.

## Research registration unique identifying number (UIN)

1. Name of the registry: Dow University Hospital.
2. Unique identifying number or registration ID: (UIN:DUH/2021/053).
3. Hyperlink to your specific registration (must be publicly accessible and will be checked): https://www.duhs.edu.pk/departments/research/downloads/IRB%20Form-20150410.doc.

## Guarantor

Muhammad Sohaib Asghar.

## Data availability statement

The data that support the findings of this study are available from the corresponding author upon request.

## Provenance and peer review

Externally peer-reviewed, not commissioned.
